# A Minimally Invasive Technique for the Management of Physiologic Gingival Melanin Hyperpigmentation: A Case Series

**DOI:** 10.7759/cureus.56511

**Published:** 2024-03-19

**Authors:** Lavanya S, Deepak M Ravindran, Santhanakrishnan Muthukumar, Balaji SK, Dhanadivya Krishnakumar

**Affiliations:** 1 Department of Periodontology, Sri Ramachandra Dental College and Hospital, Sri Ramachandra Institute of Higher Education and Research, Chennai, IND; 2 Department of Periodontology and Implantology, Sri Ramachandra Dental College and Hospital, Sri Ramachandra Institute of Higher Education and Research, Chennai, IND

**Keywords:** pink esthetics, smile correction, periodontics, esthetics, atraumatic dentistry, periodontal plastic surgery, minimally invasive dentistry, oral mesotherapy, vitamin c, gingival depigmentation

## Abstract

An attractive smile enhances an individual's self-confidence. The overall harmony of a smile can be attributed to the interplay of the teeth's shape, color, and position along with the gingival tissue. Gingival pigmentation is observed across all human races, exhibiting variations from one race to another. Typically, gingival hyperpigmentation results from the abnormal buildup of melanin in the gingival tissue, imparting a dark appearance on the gums. Various procedures, collectively known as gingival depigmentation, are employed to address gingival hyperpigmentation. While the initial outcomes of depigmentation procedures are often promising, one common issue associated with them is the potential for re-pigmentation. This article aims to evaluate the clinical effectiveness and patient-reported outcomes of intraepidermal (oral mesotherapy) vitamin C injection for nonsurgical management of physiologic gingival melanin hyperpigmentation.

## Introduction

A key objective in dentistry is to create an appealing smile, focusing on both "white esthetics" and "pink esthetics." Melanin pigmentation in the gingiva is a common occurrence that is often considered an esthetic concern, particularly in individuals with elevated smile lines and high gingival display [1].

The selection of a particular gingival depigmentation technique is influenced by factors such as the gingival biotype, the clinician's skill, and the preferences of the patient. A variety of treatment modalities have been used for removing hyperpigmentation, which involves surgical scalpel technique, laser, gingival autograft, electrosurgery, cryotherapy, and bur abrasion [2,3]. In addition, some studies have suggested that ascorbic acid (vitamin C) can be used in the management of gingival pigmentation.

Vitamin C (ascorbic acid) has shown encouraging outcomes in skin depigmentation by inhibiting tyrosinase activity [4], resulting in a decrease in dopaquinone formation, which is a precursor in melanin synthesis [5,6].

The original mesotherapy technique introduced by Pistor in dermatology [7] has undergone modifications as pioneered by Yussif et al., leading to innovations such as oral mesotherapy [8]. This method involves the administration of therapeutic agents into the intraepithelial layers of oral tissue with fine needles [9]. In dermatology, the mesotherapy technique using vitamin C has been widely reported [10], but its efficacy as a depigmenting agent for the gingiva remains an area with minimal documentation.

The objective of this case series is to evaluate the clinical efficacy and patient-related outcome measures of locally administrating vitamin C injection for minimally invasive management of physiologic gingival melanin hyperpigmentation.

## Case presentation

Case 1

**Figure 1:**
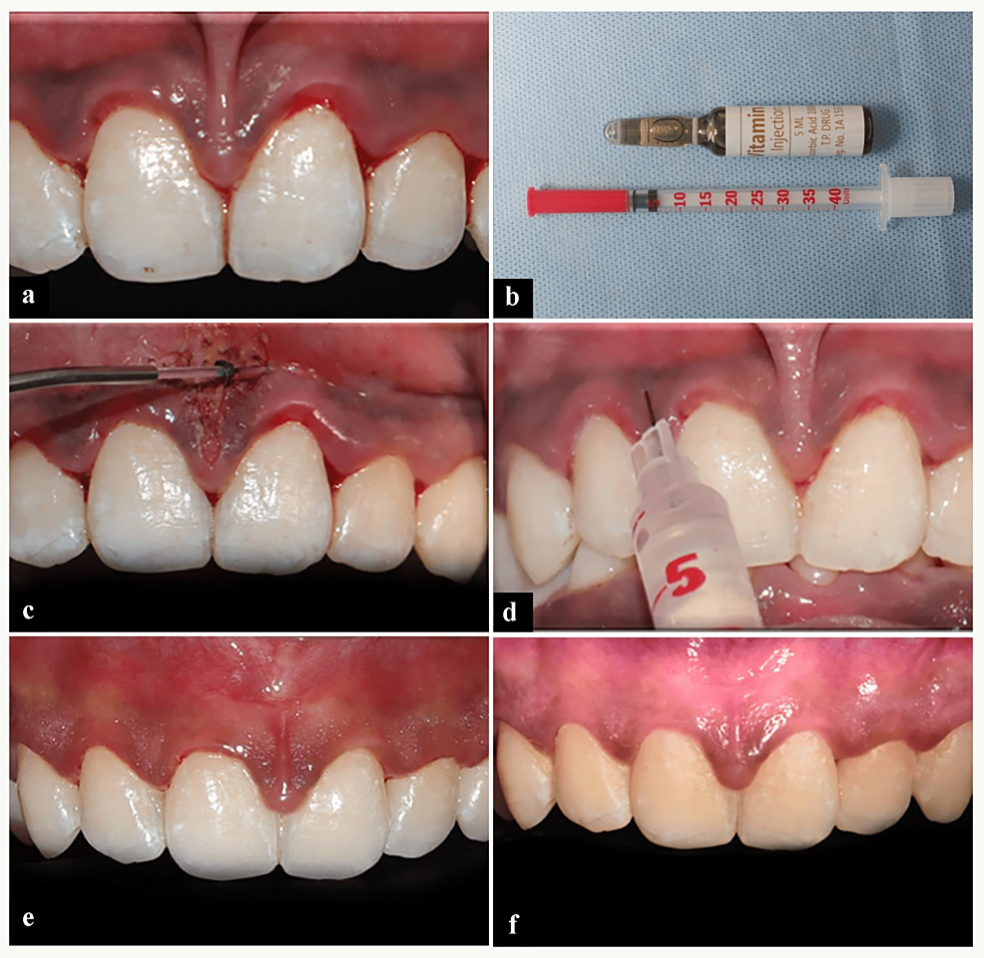
Case 1: Sequence of gingival depigmentation using ascorbic acid. (a) Preoperative image. (b) Insulin administrating syringe and vitamin C vial. (c) Frenectomy done using 940 nm diode laser. (d) Intraepithelial injection of vitamin C. (e) One-month follow-up. (f) Six-month follow-up.

A young patient aged 22 years reported to the Department of Periodontology and Implantology, Sri Ramachandra Dental College and Hospital, Chennai with the chief complaint of black spots on the gums, which looked unappealing while smiling and requiring treatment for gingival hyperpigmentation. On intraoral examination, it was found that the patient had diffuse medium-brown pigmentation in both the upper and lower gingiva. The patient was explained about the depigmentation procedures using vitamin C. Full mouth oral prophylaxis and oral hygiene instructions were given. An intraoral clinical photograph was taken before the beginning of the procedure. Initially, a topical anesthetic agent (lignocaine spray) was applied to anesthetize the pigmented area. Next, an intraepidermal gingival injection, utilizing the oral mesotherapy technique, was performed. Each injection consisted of 1.5 to 2.0 mL of vitamin C with a concentration of 250 mg (Vesco Pharmaceutical, Bangkok, Thailand). For precise drug delivery, a syringe employed for insulin administration with a 30-gauge needle was used (Figure 1).

To ensure intraepidermal administration, the needle was inserted at a depth of 0.5 to 2.0 mm. Ascorbic acid (0.1 mL) was approximately administered locally at points spaced 2 to 3 mm apart until gingival blanching occurred. The same procedure was repeated once in four weeks [11,12]. Post-treatment instructions were provided to the patient, advising them to abstain from consuming acidic, spicy, or colored foods after the procedure. Depigmentation was carried out using locally injectable vitamin C and re-pigmentation was evaluated at one-, three- and six-month intervals (Figure 1).

Case 2

**Figure 2:**
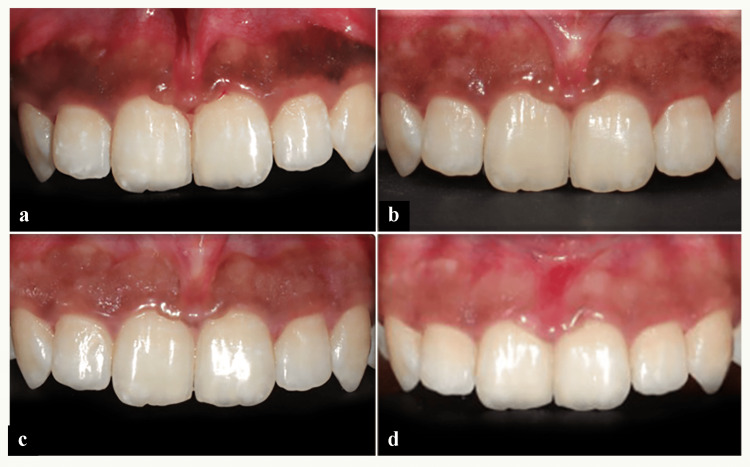
Case 2: Sequence of gingival depigmentation using ascorbic acid. (a) Preoperative image. (b) Reduction of gingival depigmentation post vitamin C injection. (c) Two-week follow-up. (d) Six-month follow-up.

A male patient aged 23 years reported chief complaints of visible black spots on upper gums. On intraoral examination and patient’s history, it was diagnosed as physiologic gingival melanin hyperpigmentation. After phase I therapy, intraepidermal administration of vitamin C was started as described earlier. This was repeated once for every four weeks. Re-evaluation was done at one-, three- and six-month intervals (Figure 2).

Case 3

**Figure 3:**
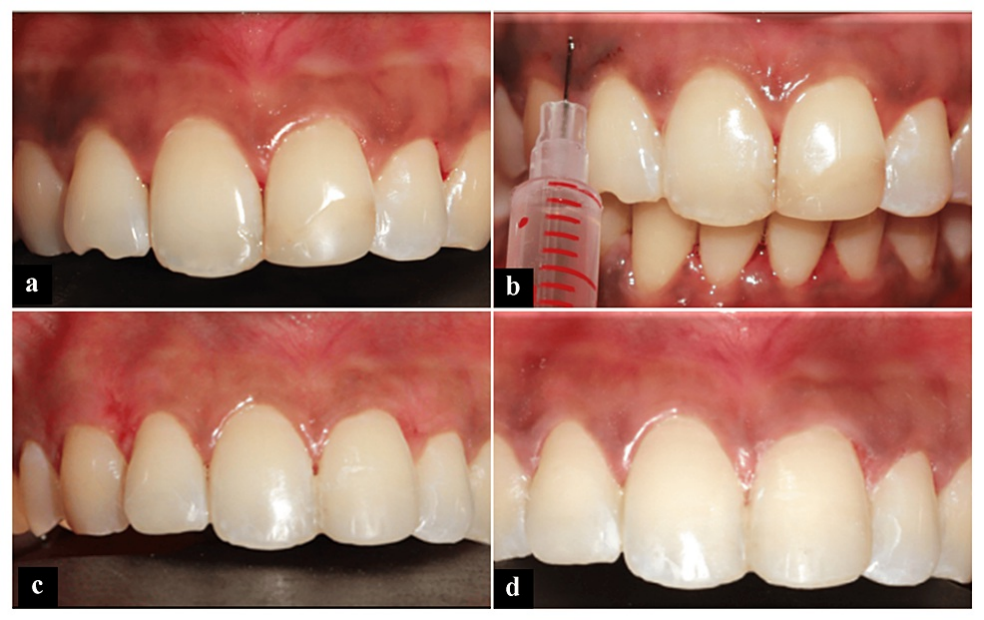
Case 3: Sequence of gingival depigmentation using ascorbic acid. (a) Preoperative. (b) Intraepithelial injection of vitamin C. (c) One-month follow-up. (d) Six-month follow-up.

A patient with physiologic gingival hyperpigmentation reported to the department requiring treatment for the removal of black spots on gums. After a thorough medical history and clinical examination, gingival depigmentation was carried out using the locally injectable vitamin C technique as described above and re-evaluated at one-, three- and six-month intervals (Figure 3).

Clinical assessment

Clinical parameters such as Dummett-Gupta Oral Pigmentation Index (DOPI) [13] and Gingival Pigmentation Index (GPI) [14] were used to quantify the amount of gingival pigmentation at one, three, and six months.

Patient-related treatment outcomes

Patient-related outcome measures were noted for pain and color improvement by asking the patients to score based on the following criteria: on a scale of 0-10, the pain was scored on the day of the procedure, on the next visit, and after one week [12]. At one-, three- and six-month follow-ups, color improvement was noted on a scale of 0-4 [15,16].

## Discussion

This study, investigating the use of ascorbic acid (vitamin C) for gingival depigmentation in these cases, provides valuable findings. Parameters like DOPI and GPI showed significant improvement at one month when compared to the initial baseline. Following the injection, there was an initial darkening of tissues attributable to the interaction between vitamin C and melanin, prompting cells to expel melanin content. Subsequently, a gradual reduction in pigmentation and an enhanced pinkish color were noted during the one-, three-, and six-month follow-ups.

Gingival color did not significantly change beyond one month, indicating no further re-pigmentation post the one-month follow-up, which was consistent with the following studies. Patient-reported outcomes were encouraging, with mild pain noted on the day of the procedure and gradually decreasing in the following days.

Literature reviews on the efficacy of vitamin C as a depigmentation agent have shown promising results. In a clinical trial, Shimada et al. observed the potential of an ascorbic acid-containing gel in treating gingival melanin depigmentation [17]. A case report by Sheel et al. has shown the effectiveness of topical vitamin C as a supplementary treatment to surgical depigmentation, noting that there was an absence of recurrence at the nine-month follow-up [18]. In two clinical studies, Yussif et al. demonstrated the use of oral mesotherapy for the direct administration of vitamin C to pigmented gingival sites, which showed promising outcomes when compared with the gold standard scalpel technique. In addition to the depigmentation effect, vitamin C can also be used as an anti-inflammatory agent in gingiva [19].

El-Mofty et al. have shown encouraging results of locally administrating vitamin C when compared to the topical formulations for gingival depigmentation. Improved patient-related outcome measures were appreciated due to mild pain and lesser discomfort and there was no disruption to daily activities [20].

Limitations of the use of vitamin C as an oral mesotherapy agent include the need for multiple appointments and at least a month for a homogenous color to appear.

## Conclusions

The positive outcomes observed in this case series emphasize the effectiveness of the oral mesotherapy technique in Indian patients, which involves the use of locally administrating vitamin C, proving to be a reliable, secure, minimally invasive, and aesthetically pleasing approach for gingival depigmentation. However, treatment duration and multiple visits to the dental office could hinder patients from optimizing oral mesotherapy using vitamin C. In the future, more randomized clinical trials with large sample sizes and long-term follow-ups are essential to identify the longevity of vitamin C's effect on gingival depigmentation, and forthcoming evidence on vitamin C as an adjunct to conventional procedures is yet to be considered.
